# *Cough*: meeting the needs of a growing field

**DOI:** 10.1186/1745-9974-1-1

**Published:** 2005-08-04

**Authors:** Rubaiyat A Haque, Kian Fan Chung

**Affiliations:** 1 Airways Disease Section, National Heart & Lung Institute, Imperial College, London, UK

## Abstract

There has been a rapidly increasing volume of research undertaken in the field of acute and chronic cough at both basic scientific and clinical levels. However, until now there has been no journal dedicated to publishing work in this field. In this editorial, we introduce the new online, open-access journal entitled *Cough *which has been founded specifically for this purpose. We also review the clinical problems posed by acute and chronic cough and highlight some of the current issues that are being tackled by cough researchers.

## A new journal for a modern era

Between 2000 and 2004, the number of PubMed entries per year containing the keyword 'cough' has increased by forty percent. This partially reflects a general increase in the number of scientific publications over this period but it also represents a specific growth in interest in cough-related research. This interest spans a number of medical and scientific disciplines other than respiratory medicine, such as gastroenterology, rhinology, paediatrics, infectious disease, pharmacology, neurology, neuroanatomy, genetics, inflammation, etc. The need for a single journal that brings together clinicians and scientists in all of these disciplines with the aim of achieving a better understanding of cough-related disease has been long overdue. So it gives us great pleasure to welcome you to *Cough*. Besides being the only journal to specialise in this particular area of research, *Cough *is special for other reasons. Embracing the modern era of information technology, *Cough *is an entirely online journal. Our online submission system allows for rapid review and revision of manuscripts and when accepted, articles appear in the journal immediately. More significantly, *Cough *is an Open Access journal. All articles are freely and universally accessible without subscription. Authors of published articles retain the copyright to their work and the full text of each article is permanently archived in PubMed Central. It will be of interest to contributors that free online access can increase the impact of a paper [1].

## The problem of acute and chronic cough

Cough is a common symptom of many respiratory and non-respiratory disorders. It is useful from a diagnostic point of view to divide cough into acute (lasting less than three weeks) and chronic (lasting longer than eight weeks) types [2]. Acute cough is the most frequently reported respiratory symptom and the commonest reason for medical consultation. The majority of cases of acute cough are due to upper respiratory viral infections. The disease is self-limiting and symptoms usually subside within a week. In this situation, cough is a short-lived complaint and confers the important benefit of airway protection and mucus clearance. However, there is a huge demand for over-the-counter cough remedies (many of which have little proven clinical benefit) and the amount of consultation time taken up by this problem is difficult to ignore. The acute viral infection-induced cough is an irritation that most sufferers would rather do without and effective antitussives are needed to address this.

Chronic cough, on the other hand, is a rather different kettle of fish. It can arbitrarily be defined as lasting longer than eight weeks but by the time these patients reach the specialist cough centre, they have suffered for a median duration of four years [3]. Chronic cough serves no obvious function. Persistent coughing has a profoundly detrimental effect on quality of life and can lead to social isolation and clinical depression. It is all too easy to attribute chronic cough to being a largely psychological phenomenon when, in fact, in the majority of cases there is a treatable cause. The commonest causes are generally accepted to be gastro-oesophageal reflux disease (GORD), rhinosinusitis and asthma. However, there is a significant minority of sufferers whose cough evades all attempts at diagnosis and treatment, earning them the label of chronic idiopathic cough. Whether idiopathic cough is a real condition or simply a failure of diagnosis continues to be debated.

## Current issues in cough research

So now that we have a forum in which cough researchers of all disciplines can take part, what directions might be taken by researchers in this field? At the level of both the afferent and efferent limbs of the cough reflex, we are now beginning to better understand the physiological and pathophysiological reflex. The anatomo-physiological approach to elucidating the cough reflex has led to the description in the guinea pig of a vagal afferent nerve subtype as being essential for defensive cough [4]. The role of other airway sensory receptors might be to modulate, rather than initiate cough. Electrophysiological studies combined with immunohistochemistry are uncovering a heterogeneity of receptors and channels present on specialised receptors such as the cough receptors. Centrally, the role of neurotransmitters such as tachykinins at the level of the 'cough centre' may partially sensitise the cough reflex. This is an important observation since patients with chronic cough have an increased cough reflex sensitivity as measured by capsaicin or citric acid challenge. Such fundamental research raises the hope of an improved understanding of the cough reflex and its sensitisation and ultimately better target identification for effective antitussives.

Clinical cough research is, in many ways, in its infancy. Part of the reason for this might be, until relatively recently, the lack of availability of tools that adequately measure cough. For some time we have believed that almost all patients with chronic cough can be cured by systematically identifying and treating asthma, GORD and rhinosinusitis. Yet there is little evidence that proton pump inhibitors and nasal steroids have any real sustained benefit in the treatment of acid- and postnasal drip-associated cough. Sedating antihistamines combined with decongestants are reported to be effective in the treatment of cough due to rhinosinusitis but the benefits may be of central origin and have little to do with postnasal drip. Are our means of diagnosing these conditions adequate? Are our treatments appropriate and genuinely effective? There seems, anecdotally at least, to be a group of chronic coughers who elude diagnosis and do not respond to any specific treatment. These idiopathic coughers form a significant proportion of patients in certain cough centres [3]. These patients are likely to have a variety of as yet unidentified pathologies and are fertile ground for future investigation.

Whatever directions are taken in the future by cough researchers, the journey will no doubt be an exciting one. The launch of *Cough *will help to keep us all better informed of developments in this area and assist those in the field in directing research endeavours. We would like to thank the members of our Editorial Board for their willingness to contribute both their time and expertise to this project. We are all, of course, indebted to the staff at BioMed Central for their invaluable assistance with this launch.

## Competing interests

A small proportion of article processing charges (APC) from accepted manuscripts goes to the editorial office to help with the running costs of the journal. Additional costs have been met by an unconditional grant from AstraZeneca.

## References

[B1] Lawrence S (2001). Free online availability substantially increases a paper's impact. Nature.

[B2] Chung KF, Widdicombe J (2004). Acute and chronic cough. Pulm Pharmacol Ther.

[B3] Haque RA, Usmani OS, Barnes PJ (2005). Chronic Idiopathic Cough: A Discrete Clinical Entity?. Chest.

[B4] Canning BJ, Mazzone SB, Meeker SN, Mori N, Reynolds SM, Undem BJ (2004). Identification of the tracheal and laryngeal afferent neurones mediating cough in anaesthetized guinea-pigs. J Physiol.

